# Integrated transcriptomics approach identifies the upregulated SLC7A5, and associated pathways across different thyroid cancer cell types

**DOI:** 10.3389/fgene.2026.1890433

**Published:** 2026-09-03

**Authors:** Muhammad Naeem, Nan Wu, Yang Wu, Shahid Nawaz, Ren Jing, Yuanbin Luo, Shijian Yi

**Affiliations:** 1 Department of Breast and Thyroid Surgery, South China Hospital, Medical School, Shenzhen University, Shenzhen, China; 2 Guangdong Key Laboratory for Biomedical Measurements and Ultrasound Imaging, National-Regional Key Technology Engineering Laboratory for Medical Ultrasound, School of Biomedical Engineering, Shenzhen University, Medical School, Shenzhen, China; 3 Centre for Applied Molecular Biology, University of the Punjab, Lahore, Pakistan

**Keywords:** DEGs, differential expression, KEGG, PPI network, SLC7A5, thyroid cancer

## Abstract

Thyroid cancer is the most common type of endocrine malignancy, and its aggressive types are diagnosed at advanced stage due to limited treatment options. This study aimed to identify upregulated SLC7A5 across thyroid cancer cell types using an integrated transcriptomics approach. Total RNA was extracted from different samples. Differential expression analysis was performed through DESeq2. Functional enrichment analyses were performed to explore key pathways. The PPI network was built using the STRING database to investigate the functional relationships among significantly differentially expressed genes. Differential expression analysis revealed that SLC7A5 was significantly upregulated in KTC-1. GO and KEGG analyses were enriched with cell adhesion, protein binding, extracellular exosome, RNA processing, ECM-receptor interactions, and focal adhesion. The PPI network analysis showed the interaction of SLC7A5 with TERF1, CARD10, PSAT1, SIRPA, and MYC. Western blot analysis revealed that expression of mTOR was not elevated in KTC-1. Overall, these results could provide valuable insights for further validation in thyroid cancer therapy.

## 1 Introduction

Thyroid cancer is the most common type of endocrine malignancy, and more than 237,000 cases were reported worldwide in 2022. It has become a global health issue due to the increasing number of cases in recent years (Li et al., 2026). Limited treatment options are available for early-stage detection of thyroid cancer (Lee et al., 2026). However, the risk of complications increases due to limited treatments. Based on histological features and cell origin, it is classified into different types, such as papillary, anaplastic, and follicular thyroid cancers. It was observed that patients with anaplastic thyroid cancer were diagnosed at an advanced stage, with a median 1-year survival rate about 10–20% (Moreno et al., 2022). Some patients with advanced stages of differentiated thyroid cancers could develop radioiodine-refractory disease (RAIR) (Hosseinzadeh et al., 2025). Therefore, early stage detection and precise treatment are crucial for improving survival rates in thyroid cancer patients (Codrich et al., 2025).

Previous literature reported that some genes have been identified in the development of thyroid cancer (Vaio et al., 2026). Mutations in the *RAS* (rat sarcoma virus) and *BRAF* (v-raf murine sarcoma viral oncogene homolog B) genes had been reported in papillary thyroid cancer (Moposita Molina et al., 2025). *PLAU* (Plasminogen activator), *COL1A1* (Alpha type I collagen protein), *RRM2* (Ribonucleoside reductase subunit M2), and *LGALS3* (Galectin-3) had reported in papillary thyroid cancer (Sarin et al., 2022; Qiu et al., 2016). *HRAS* (Harvey rat sarcoma homolog)*, KRAS* (Kirsten rat sarcoma homolog), and *NRAS* (neuroblastoma proto-oncogene) genes were observed in the progression of follicular thyroid cancer (Chiba, 2024). Moreover, *TERT* (telomerase reverse transcriptase), *PIK3CA* (phosphatidylinositol 3-kinase catalytic subunit alpha), and *PTEN* (phosphatase and tensin homolog) also reported in anaplastic thyroid cancer (Cleere et al., 2024).

SLC7A5 is a membrane transporter involved in cellular signaling, protein synthesis, amino acid transport, and physiological processes (Jeckelmann et al., 2022). Different roles of SLC7A1, SLC7A5, SLC7A7, and SLC7A11 observed in breast, thyroid, and ovarian cancers. SLC7A1 was reported in arginine uptake for promoting the tumor growth in patients with ovarian cancer. Its expression was high in tumor tissues relative to the control group (Gong et al., 2021). SLC7A5 participates in leucine uptake, which is involved in the mTOR signaling pathway (Sokolov et al., 2020). Wang et al. (2024) reported that SLC7A7 stimulates cell growth and proliferation in colorectal cancer (Wang et al., 2024). SLC7A11 stimulates glutathione (GSH) synthesis via cystine-glutamine exchange and protects thyroid cancer cells from ferroptosis (Wang et al., 2021). However, limited literature is available on the role of SLC7A5 in thyroid cancer.

RNA sequencing technology is used to identify novel genes, alternative splicing events, and gene alterations. This approach provides the precise measurement of transcript expression analysis (Pan et al., 2024). Integration of RNA sequencing data with clinical datasets could provide a platform for categorizing the novel DEGs into biological processes, cellular components, and molecular functions associated with cell cycle regulation, metabolic reprogramming, DNA replication and RNA processing, mTOR signaling, biosynthetic metabolism, and cellular growth. However, the upregulation of SLC7A5 and its involvement in cellular processes and signaling pathways remain unclear (Byun et al., 2025; Xu T. et al., 2025).

SLC7A5 is a membrane transporter involved in mTOR signaling, supporting its biological and translational relevance for prioritizing significantly differentially expressed genes. In this study, we performed an integrated RNA-seq analysis to identify SLC7A5 upregulation across different thyroid cancer cell lines. We also performed functional enrichment analyses to identify the biological processes, cellular components, and molecular functions. The PPI network was built to investigate the functional relationships among differentially expressed genes. Western blot analysis was performed to evaluate expression levels.

## 2 Methods

### 2.1 Chemicals and reagents

RPMI-1640 was obtained from Thermo Fisher Scientific (New York, USA). Trizol was purchased from Apexbio Technology (Houston, USA). Fetal bovine serum (FBS) was utilized for cell growth and supplied by Excell Biotechnology (Suzhou, China). HRP secondary antibody was obtained from Jackson ImmunoResearch Company (Pennsylvania, USA). Phosphate-buffered saline (pH∼7.4) was used for cell washing and obtained from Biyun Tian Biotechnology (Shanghai, China).

### 2.2 Cell cultivation

KTC-1 cells (Catalog No. ZQ0318) were purchased from Zhong Qiao Xin Zhou Biotechnology Co., Ltd., Shanghai, China. These cells referred to a well-characterized human thyroid cancer cell model derived from an adult patient with poorly differentiated papillary thyroid cancer and originated from the site of metastatic pleural effusion. ATC, FTC, KTC-1, and Nthy-ori 3-1 were cultured in RMIP-1640 with the addition of 10% FBS and 1% penicillin. The reaction mixture was incubated with 5% CO_2_ at 37 °C for 24 h. Trizol reagent was added for total RNA extraction, and samples were stored at −80 °C for further analysis.

### 2.3 RNA extraction, and RNA sequencing analysis

Total RNA was extracted from ATC, FTC, and KTC-1 using an RNA extraction kit, and purity was determined using the Agilent 5300 Fragment Analyzer (Agilent, CA, USA). Capture beads were used for mRNA purification, and fragmentation was performed at 94 °C in the presence of magnesium ions (cat. no. 12340ES97; Shanghai Yeasen Biotechnology Co., Ltd). The cleaved RNA fragments were reverse-transcribed to produce the first cDNA strand. Each strand was modified by adding an A base pair to an adapter containing a T base pair. Amplification was performed using a thermocycler, and PCR products were purified with Hieff NGSDNA selection beads. Sequencing analysis was performed using the Illumina Novaseq™ X Plus (LC-Bio Technology CO., Ltd., Hangzhou, China).

### 2.4 Workflow of integrated RNA-seq transcriptomics

**FIGURE 1:**
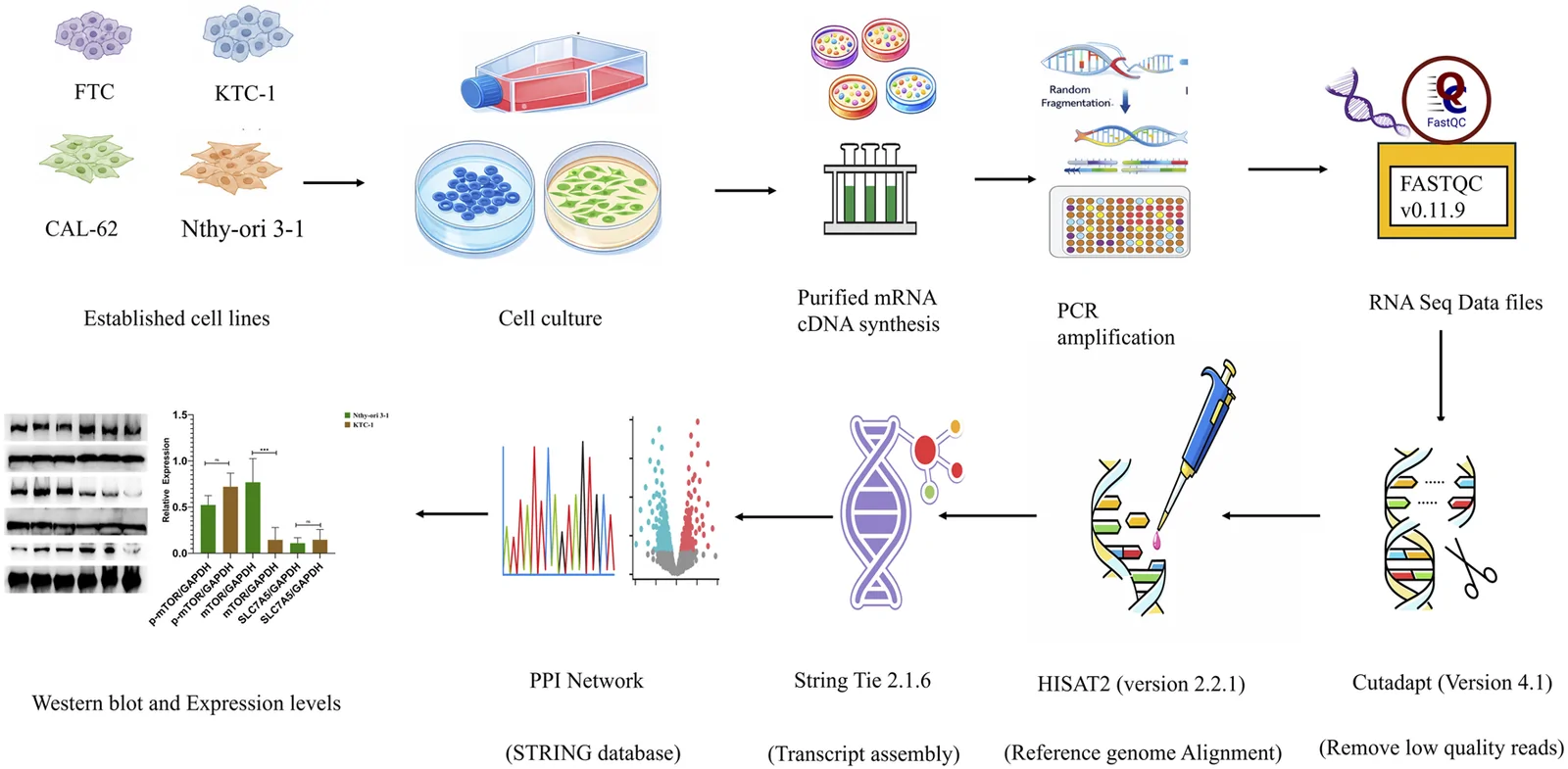
Integrated RNA-seq transcriptomic approach to identify the key signaling pathways, and upregulated SLC7A5. Firstly, different thyroid cancer cells (ATC, FTC, and KTC-1) and normal thyroid epithelial cells were cultured. Total RNA was extracted, followed by RNA fragmentation, cDNA synthesis, PCR amplification, and sequencing analysis. Raw sequencing reads were evaluated for quality control, followed by adapter removal, elimination of low-quality reads, read alignment to a reference genome, transcript reconstruction, differential gene expression analysis, and functional enrichment analysis.

Raw sequencing reads were evaluated for quality control, followed by the removal of low-quality reads, reference genome alignment, transcript reconstruction, gene expression quantification, differential gene expression analysis, and functional enrichment analysis (Figure 1).

### 2.5 Raw read quality control and pre-processing analysis

FastQC (v0.11.9) was used for assessment of GC content, sequence quality, adapters contamination, and sequence repetition. MultiQC (v1.14) was used to summarize the quality control reports. Reads having a minimum length were eliminated after trimming. This process ensured that only high-quality and sufficient reads were retained for downstream analysis.

### 2.6 Reference genome alignment

HISAT2 (version 2.2.1) was used for mapping the post-processed data into the human reference genome (GRCh38/hg38). Alignment files were generated in BAM format using HISAT2. Alignment quality was evaluated by measuring key metrics and the total number of reads.

### 2.7 Transcript reconstitution and expression measurement

StringTie (version 2.1.6) was used for transcript reconstruction and expression measurement. Gffcompare tool (version 0.9.8) was used for construction of comprehensive transcriptome across samples. Levels of gene expression were assessed in FPKM units for normalization of sequencing depth, and transcript length. StringTie software was used for calculation of the FPKM values.

### 2.8 Gene-level expression analysis

Quantification was performed at the gene level, and reads counts were independently normalized to obtain the FPKM. The transcript-level expression data were analyzed using isoform-specific information, and downstream analyses in the present study were focused on gene-level expression, which was sufficient to address the biological questions of interest.

### 2.9 Annotation of transcriptomes and other RNA species

Oligo(dT) enrichments were performed for the generation of sequencing libraries. Each gene has annotation information, location of chromosome, codon positions, read counts, GO, and KEGG. Other classes of RNAs were further investigated using transcript-type annotations in the reference genome.

### 2.10 Differential gene expression analysis

DESeq2 (v1.38.3) was used to perform differential gene expression analysis. The low-expressed genes were removed due to weak expression signals. Wald test was applied for differential gene expression analysis. Genes that passed the |log_2_FC| ≥1, and q-value <0.05 (the q-value is the adjusted p-value) were considered differentially expressed.

### 2.11 Heatmap and principal component analysis

Principal component analysis (PCA) was performed for assessment of transcriptomics correlations of different samples. The heatmap of top 100 DEGs was constructed using the pheatmap (v1.0.12). Normalization was performed using Z-score, and Euclidean distance was used for hierarchical clustering analysis.

### 2.12 Functional enrichment analysis

ClusterProfiler (v4.6.2) was used for assessment of GO and KEGG analyses of DEGs. Benjamini-Hochberg method was used for determination of enrichment significance. Reactome pathways were analyzed using the ReactomePA (v1.42.0). Gene sets (upregulated and downregulated) were examined independently to identify key pathways in thyroid cancer cells.

### 2.13 Protein-protein interaction (PPI) analysis

The protein-protein interaction (PPI) network analysis was constructed using the STRING database to investigate the functional relationships among differentially expressed genes.

### 2.14 Western blot analysis

Total proteins were extracted in the presence of RIPA lysis buffer and separated by SDS-PAGE. The PVDF membranes were used for transferring the resolved proteins, blocked with 5% skimmed milk, and incubated with primary and secondary antibodies. The phospho-mTOR antibody was used to detect the phosphorylation site at Ser2481.Western blot experiments were performed with three biological replicates, and data were presented as mean ± SD. ImageJ software was used for densitometry analysis of blots (Supplementary Table 1). Relative protein expression was normalized to GAPDH. Statistical analysis was performed using the Sidak test, and significance was set as p < 0.05. The expression profiles of different proteins were evaluated using a high-sensitivity chemiluminescence kit (Kangwei, Beijing, China). Different antibodies were used, such as p-mTOR (Catalog No: bs-3495r, Bioss, Woburn, United States), mTOR (Catalog No: 66888-1-Ig, Proteintech, Wuhan, China), SLC7A5 (Catalog No: YP-mAb- 09823, UpingBio, Hangzhou, China ), GAPDH (Catalog No: ET1601-4, Huabio, Massachusetts, USA), and HRP-Goat anti Rabbit (Catalog No: 111-035-003, Jackson, Pennsylvania, United States).

### 2.15 Statistical analysis

Benjamini-Hochberg method was used for determination of enrichment significance. Functional enrichment analyses were performed for the identification of key pathways using the false discovery rate (FDR) method. Sidak test was performed for multiple comparisons.

## 3 Results

### 3.1 Transcriptomic differences between thyroid cancer and normal cells

**FIGURE 2:**
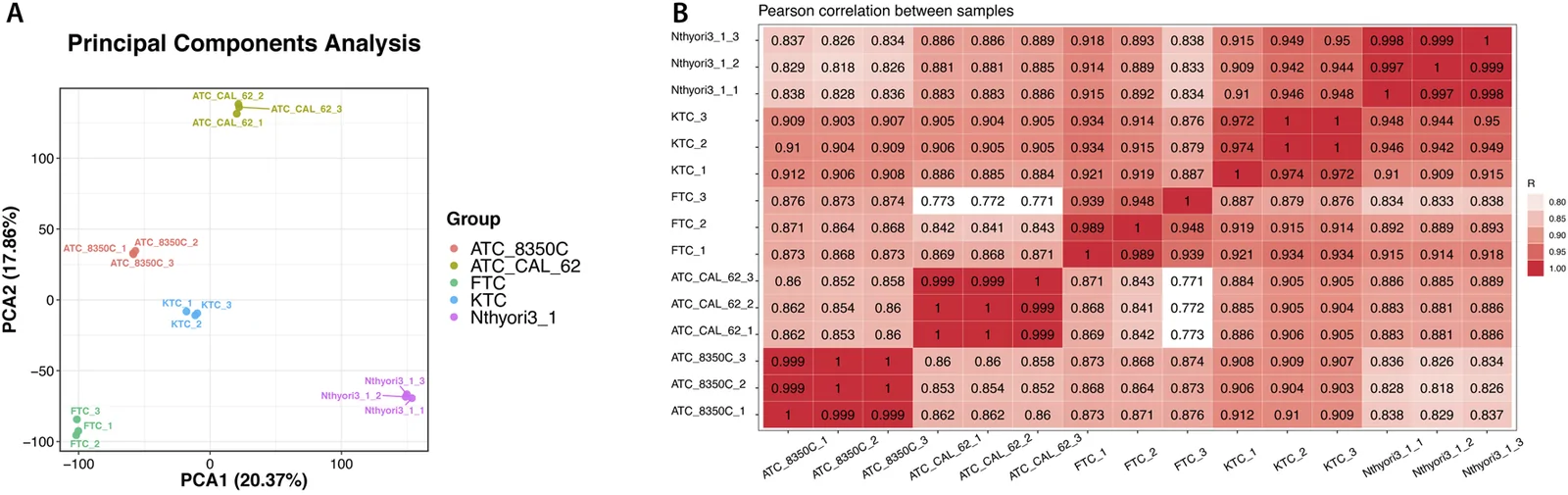
Transcriptomic differences distinguish the thyroid cancer cell types from normal thyroid cells. **(A)** Principal component analysis (PCA) of RNA-seq expression profiles of ATC, FTC, and KTC-1 and normal samples. PCA clearly separates cancer and normal samples, indicating extensive transcriptomic remodeling. Normal thyroid samples show tight clustering, reflecting transcriptional homogeneity, whereas thyroid cancer samples segregate by type. **(B)** The sample-to-sample distance heatmap shows the strong clustering within biological replicates and clear separation between normal and cancer cells.

Principal component analysis (PCA) of the top variable genes revealed distinct differences between thyroid cancer and normal cells along PCA1 and PCA2, indicating extensive transcriptomic reprogramming (Figure 2A). ATC cells were furthest from normal human thyroid epithelial cells along the principal components. FTC and KTC-1 cells were separated into intermediate clusters between ATC and normal cells. The sample-to-sample distance heatmap shows strong clustering within biological replicates and clear separation between thyroid cancer cells and normal human thyroid epithelial cells (Figure 2B).

### 3.2 Differential gene expression analysis identifies extensive transcriptional reprogramming

**FIGURE 3:**
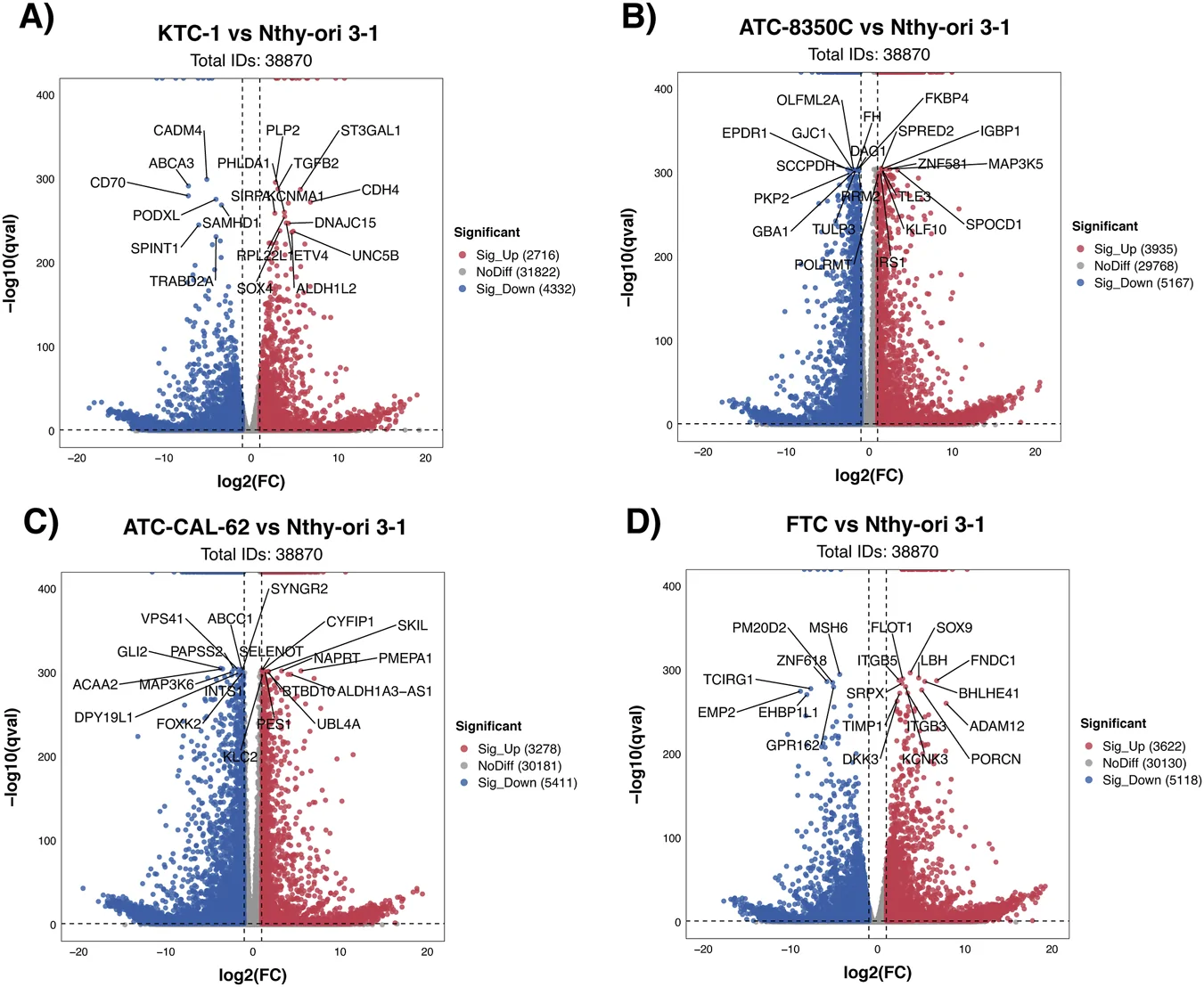
Volcano plot showing the top 20 differentially expressed genes. **(A)** KTC-1 vs. Nthy-ori 3-1 **(B)** ATC-8350C vs. Nthy-ori 3-1 **(C)** ATC-CAL-62 vs. Nthy-ori 3-1 **(D)** FTC vs. Nthy-ori 3-1. Each point represents a gene, plotted by log_2_ fold change.

The volcano plot for each thyroid cancer cell shows upregulated and downregulated genes. The volcano plot of KTC-1 shows 2716 upregulated and 4332 downregulated genes. PLP2, ST3GAL1, TGFB2, CDH4, DNAJC15, UNC5B, and ALDH1L2, were significantly upregulated. The volcano plot for ATC-8350C shows 3935 upregulated and 5167 downregulated genes. SPRED2, IGBP1, MAP3K5, SPOCD1, and TLE3 were significantly upregulated. Moreover, the volcano plot for FTC shows 3622 upregulated and 5118 downregulated genes. SOX9, LBH, FNDC1, BHLHE41, ADAM12, and PORCN genes were significantly upregulated (Figures 3A–D).

**FIGURE 4:**
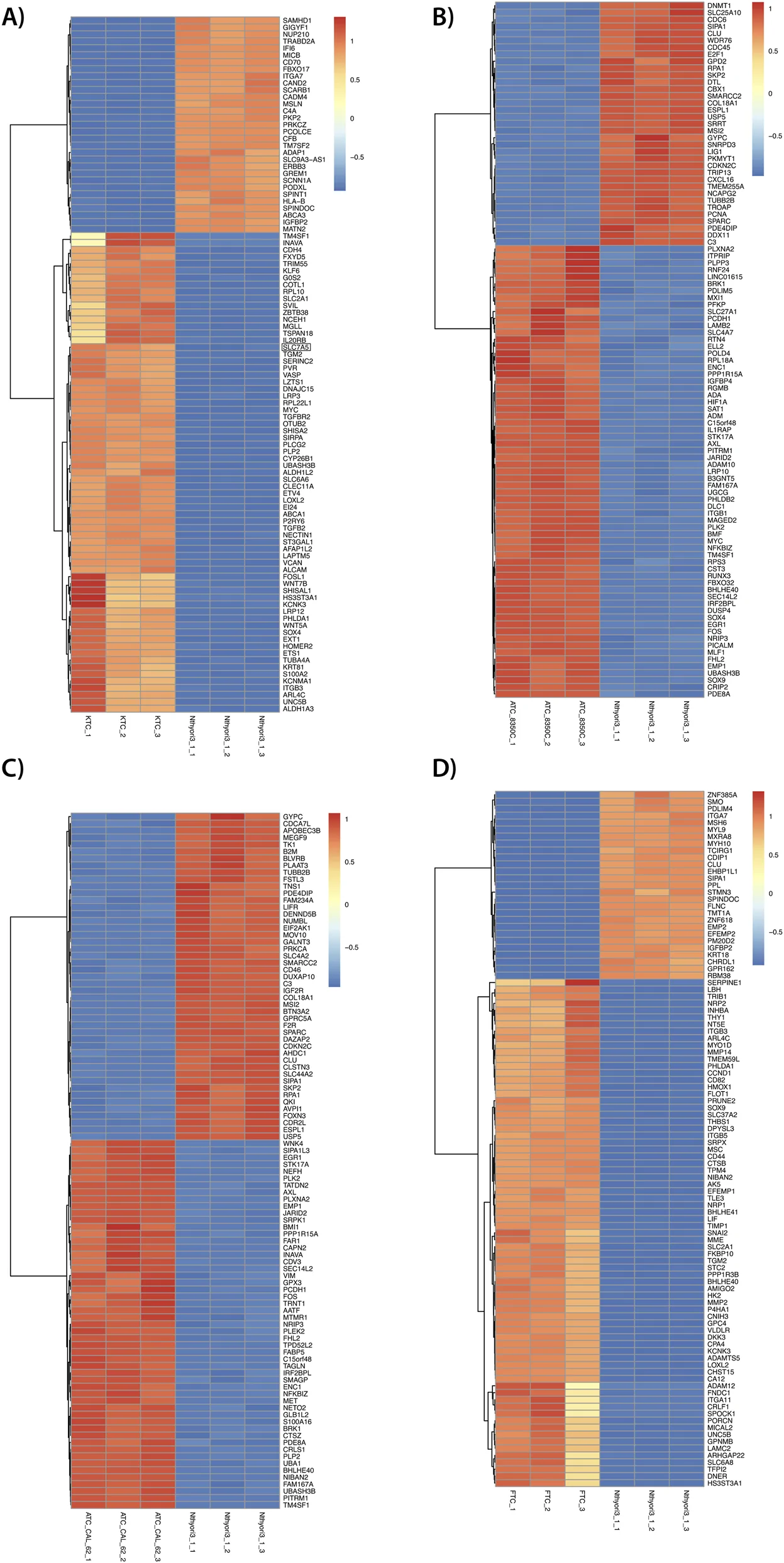
Heatmap representing the top 100 differentially expressed genes (DEGs). **(A)** KTC-1 vs. Nthy-ori 3-1, showing distinct transcriptional reprogramming in KTC-1. SLC7A5 is highlighted in circle and included among significantly upregulated genes. **(B)** ATC-8350C vs. Nthy-ori 3-1 **(C)** ATC-CAL-62 vs. Nthy-ori 3-1 **(D)** FTC vs. Nthy-ori 3-1. Rows represent the genes, and columns represent individual samples. Color intensity reflects the relative gene expression levels (red, upregulation; blue, downregulation). The heatmaps demonstrated the type-specific transcriptional signatures.

The top 100 significantly differentially expressed genes were converted into a heatmap showing expression patterns. Upregulated SLC7A5 was highlighted in a circle as shown in Figure 4A. Therefore, its differential expression analysis was discussed in the next section.

### 3.3 Expression profiling of SLC7A5

Using the criteria of |log_2_FC| ≥ 1, and q-value <0.05 (the q-value is the adjusted p-value), we found that SLC7A5 gene expression was significantly upregulated in KTC-1. Therefore, SLC7A5 was selected for further study due to its significant expression (Figure 4A; Supplementary Table 2). Differential expression of SLC7A5 was not observed in ATC and FTC as compared to normal human thyroid epithelial cells (Figures 4B–D).

### 3.4 Functional enrichment analysis and associated pathways

**FIGURE 5:**
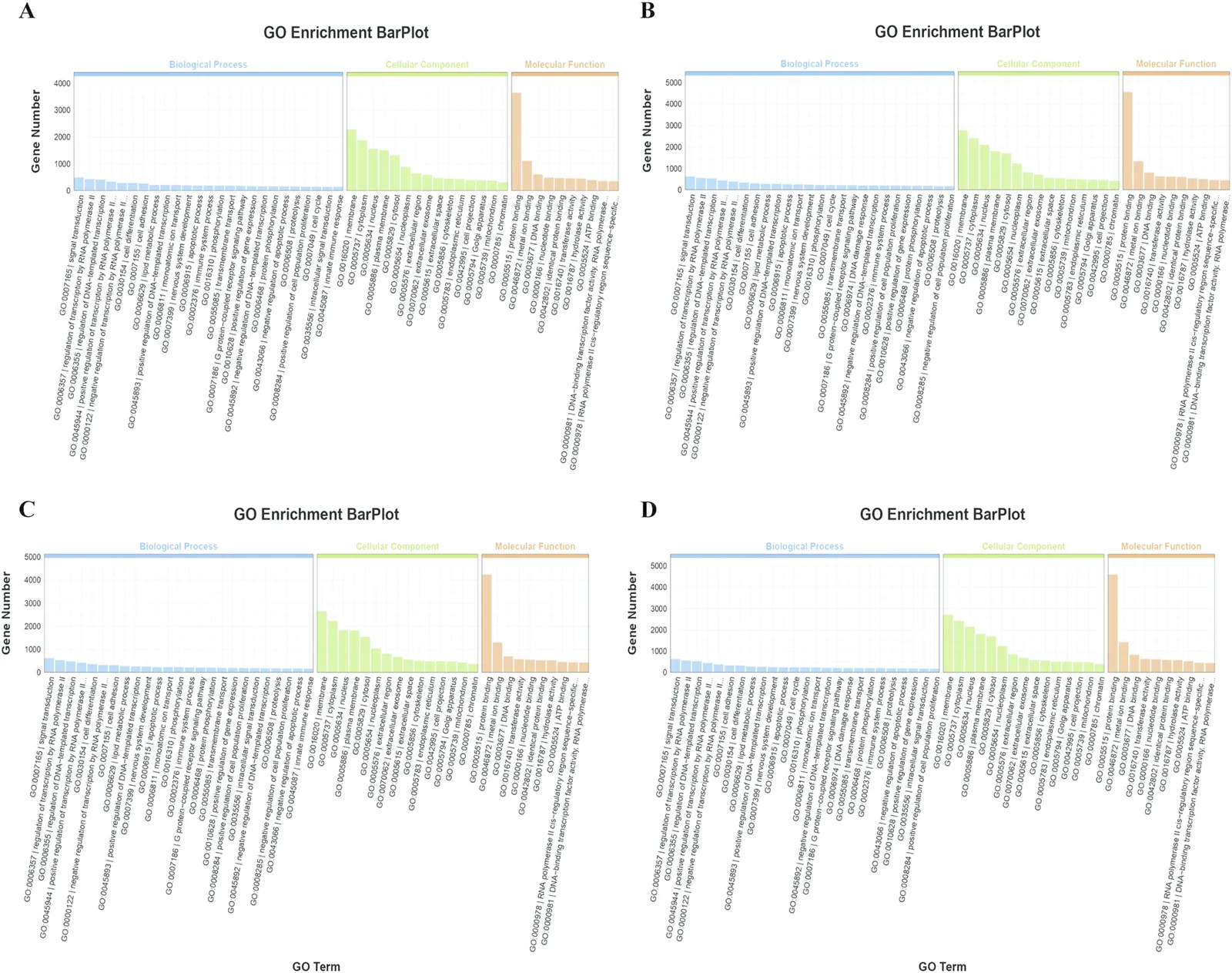
Gene Ontology (GO) biological process enrichment analysis of differentially expressed genes. **(A)** GO enrichment bar plot for KTC-1 vs. Nthy-ori 3-1. **(B)** ATC-8350C vs. Nthy-ori 3-1 **(C)** ATC-CAL-62 vs. Nthy-ori 3-1 **(D)** FTC vs. Nthy-ori 3-1.

**FIGURE 6:**
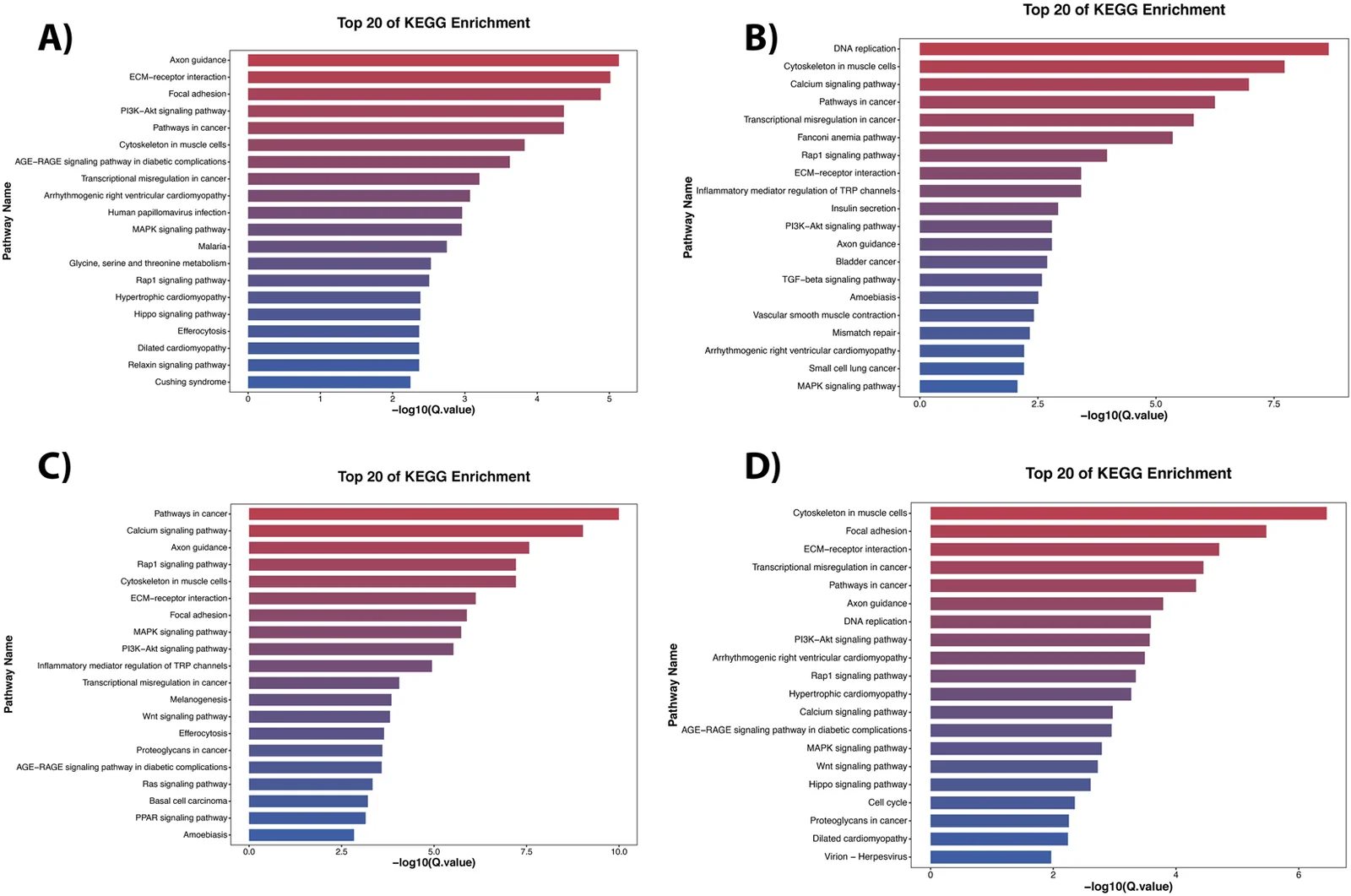
KEGG pathway enrichment analysis of differentially expressed genes. **(A)** KTC-1 vs. Nthy-ori 3-1 **(B)** ATC-8350C vs. Nthy-ori 3-1 **(C)** ATC-CAL-62 vs. Nthy-ori 3-1 **(D)** FTC vs. Nthy-ori 3-1. KEGG enrichment profiles reveal the distinct pathways in thyroid cancer cells.

**FIGURE 7:**
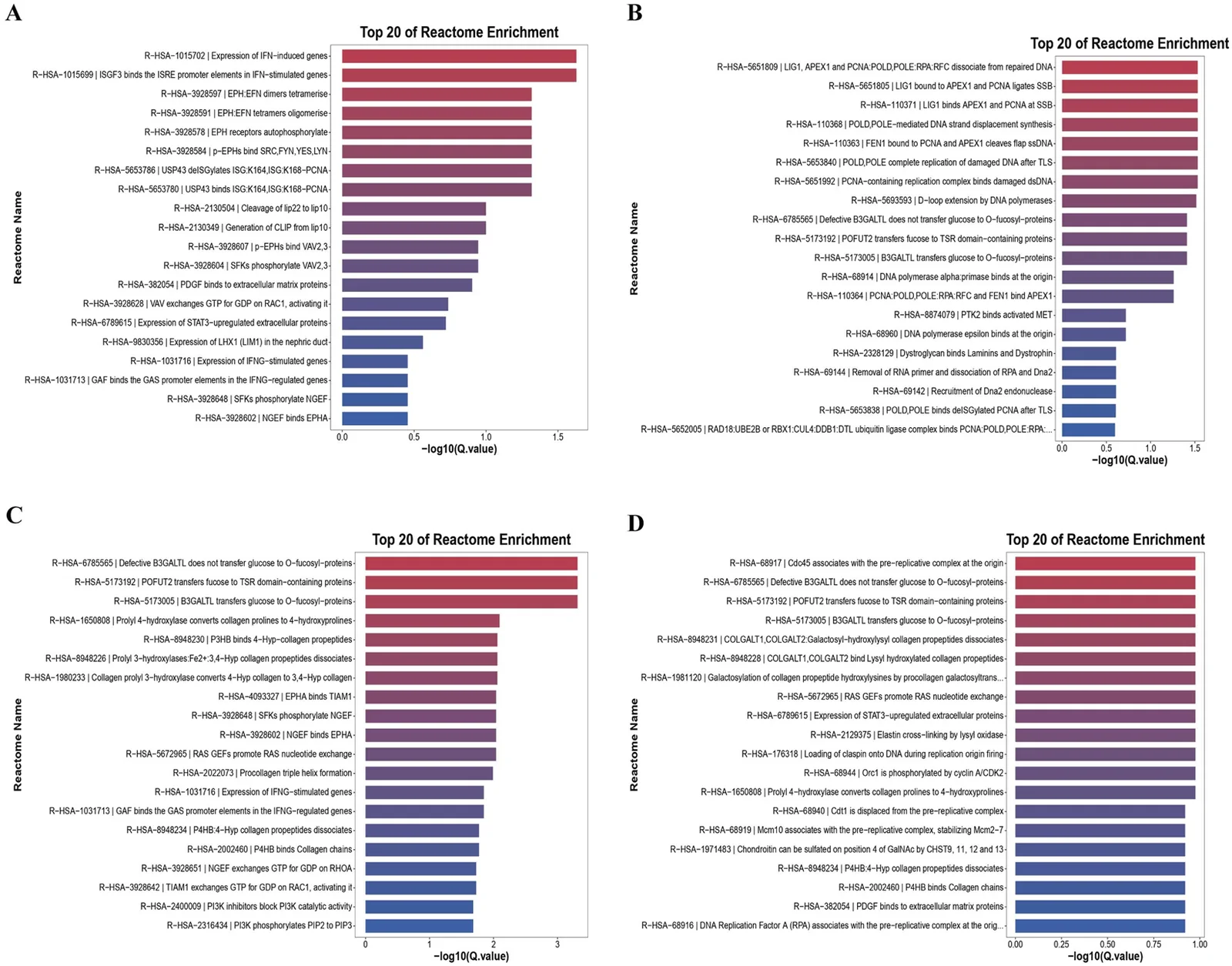
Reactome pathway enrichment analysis of differentially expressed genes. **(A)** KTC-1 vs. Nthy-ori 3-1 **(B)** ATC-8350C vs. Nthy-ori 3-1 **(C)** ATC-CAL-62 vs. Nthy-ori 3-1 **(D)** FTC vs. Nthy-ori 3-1. Reactome enrichment profiles further emphasize the type-specific pathway activation.

Enrichment analyses were performed to identify biological processes and associated pathways (Figures 5A–D, 6A–D, 7A–D). Gene Ontology (GO) enrichment analysis in KTC-1 showed that biological processes enriched with cell adhesion, protein binding, extracellular exosome, RNA processing, and transmembrane transport. KEGG analysis showed key pathways enriched with ECM-receptor interactions, focal adhesion, and PI3K-Akt. These analyses were performed on the complete set of differentially expressed genes, and SLC7A5 was associated with these biological processes and pathways. GO and KEGG enrichment analyses in ATC and FTC did not reveal pathways similar to those for SLC7A5 (Figures 5A–D, 6A–D). Reactome enrichment profiles further emphasize the type-specific pathway activation and transciptomics involvment (Figures 7A–D).

### 3.5 PPI network analysis

**FIGURE 8:**
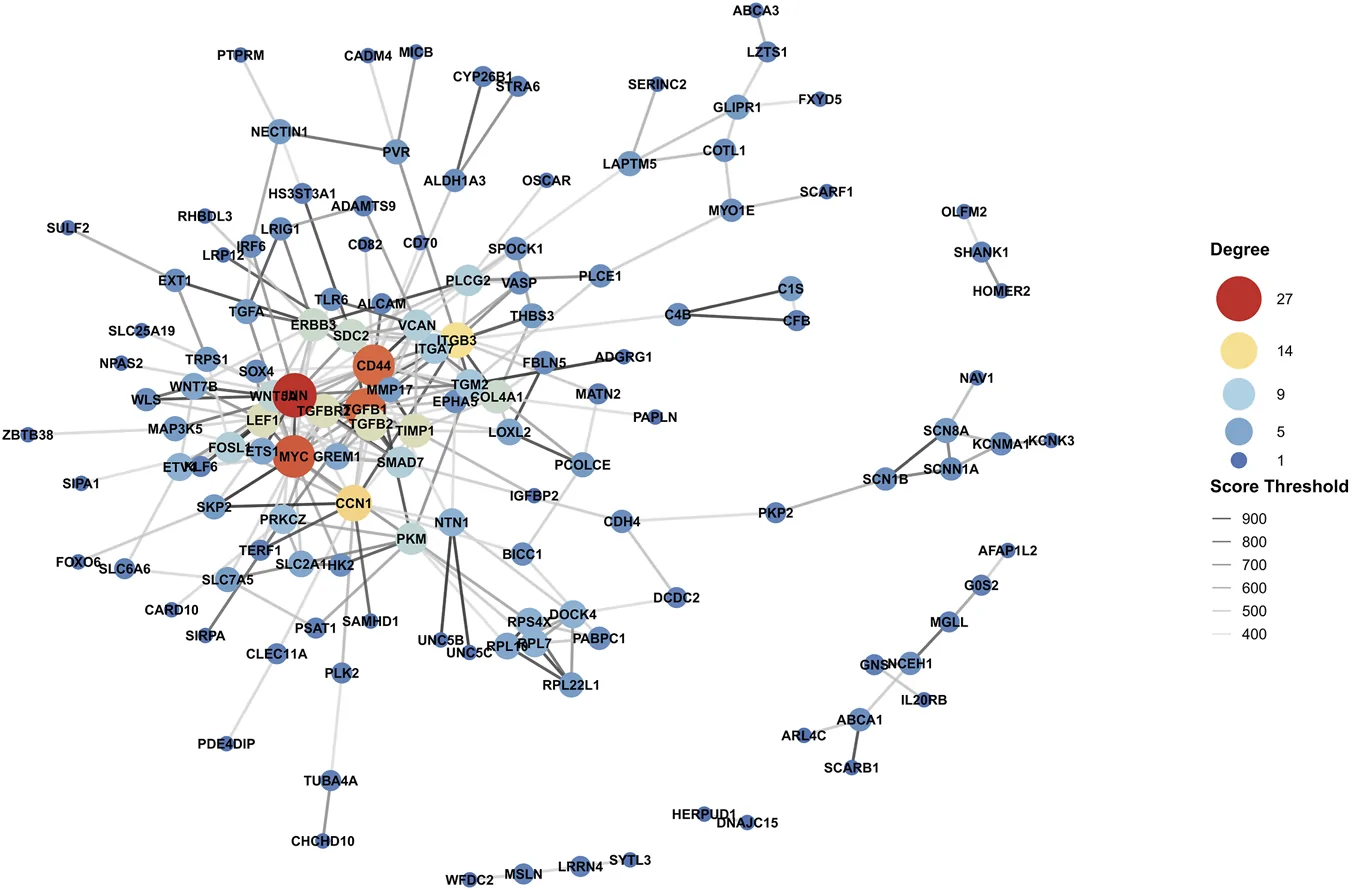
Protein-protein interaction (PPI) network created with STRING of the significantly differentially expressed genes in KTC-1 vs. Nthy-ori 3-1. Proteins are represented as nodes, and experimentally validated or predicted functional interactions are represented as edges. The node size indicates the degree of connectivity with central hub proteins. SLC7A5 is incorporated into the central interaction network, indicating its involvement in KTC-1.

A protein-protein interaction (PPI) network was built using the STRING database and functionally coordinated by forming a dense core through interconnected nodes. SLC7A5 was located within the network and interacted with various proteins, including TERF1, CARD10, PSAT1, SIRPA, and MYC (Figure 8). Cell adhesion and extracellular matrix organization proteins, such as VCAN, COL4A1, THBS3, FBLN5, ALCAM, and CD44 also interacted in KTC-1. The resultant network depicts the numerous interrelations among different proteins involved in cellular processes.

### 3.6 Western blot and expression analysis

**FIGURE 9:**
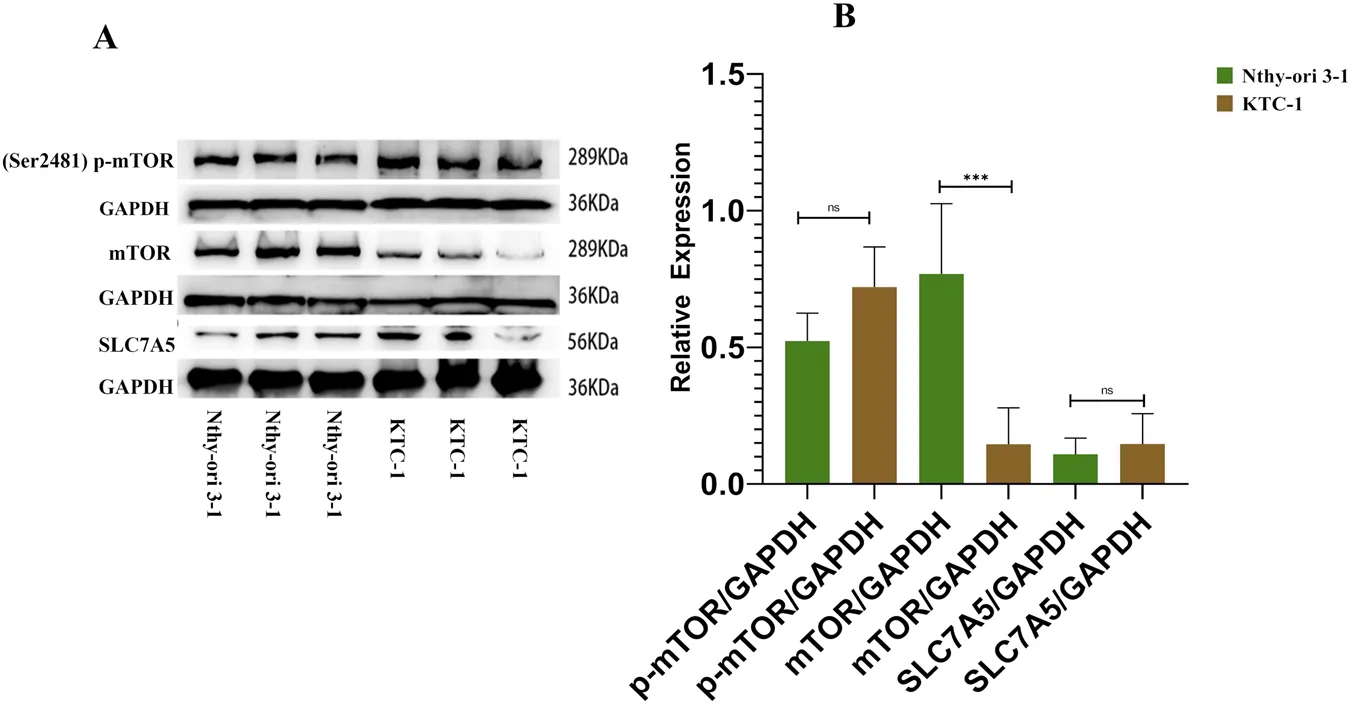
Western blot analysis and expression levels. **(A)** The results included the blot images. The phosphorylation site was detected by the phospho-mTOR antibody at Ser2481. **(B)** ImageJ software was used for densitometry analysis of blots. Relative protein expression was normalized to GAPDH. The experiments were performed with three biological replicates, and data were presented as mean ± SD. Statistical analysis was performed using the Sidak test. p < 0.001 ***(highly significant); ns: non-significant.

On the basis of the previously reported association between SLC7A5 and mTOR, Western blot analysis was performed to evaluate the expression profiles. The results revealed that expression of mTOR was not elevated in KTC-1. SLC7A5 and p-mTOR were also assessed, and the results suggested an association that requires further validation (Figures 9A,B).

## 4 Discussion

In this study, we performed an integrated transcriptomic analysis across different thyroid cancer cell lines. The expression patterns revealed the distinct transcriptional profiles for cell type. Limited treatment options are available for early-stage detection of thyroid cancer (Gil-Bernabé et al., 2025). Sun et al. (2021) reported that ENTPD1, RUNX1, THRSP, KLK10, and MIR31HG were upregulated in papillary thyroid cancer (Sun et al., 2021). Another study by Fan et al. (2021) identified the hub genes LPAR5, TFPI and ENTPD1 in papillary thyroid cancer (Fan et al., 2021). SPOCK1 is a multi-domain glycoprotein that is involved in extracellular matrix organization, and cell adhesion (Karaman et al., 2025; Xu et al., 2020). SCARF1 is a multilayer network of proteoglycans, and its altered expression has been observed in gastric cancer (Patten, 2018). DCDC2 is a structural protein involved in microtubule localization process (Dai et al., 2023). Our transcriptomic analysis revealed the significantly differentially expressed genes in KTC-1, including SPOCK1, SCARF1, and DCDC2. However, the role of these genes in thyroid cancer cells remains unclear, suggested further functional studies.

SLC7A5 is involved in cellular signaling, protein synthesis, energy metabolism, and physiological processes (Jeckelmann et al., 2022). Gong et al. (2021) reported that SLC7A1 promoted tumor growth through activating the mTOR signaling pathway in ovarian cancer (Gong et al., 2021). Chen et al. (2022) investigated that SLC7A6 contributed to angiogenesis and activated the mTORC1/S6K pathway in hepatocellular carcinoma (Chen et al., 2022). Dai et al. (2021) investigated that SLC7A7 was involved in cell proliferation and PIK/AKT pathway in lung cancer (Dai et al., 2021). Western blot analysis revealed that expression of mTOR was not elevated in KTC-1. However, expression profiles of SLC7A5 and p-mTOR were also assessed and suggested an association requiring further validation (Dunlap et al., 2025; Scalise et al., 2018).

GO analysis provides a framework for revealing the biological processes, cellular components, and molecular functions. These processes are important for a better understanding the events occurred during thyroid cancer development (Menyhart et al., 2025). Sun et al. (2021) performed GO analysis and reported that mitotic spindle organization and chromosome segregation were crucial for maintaining the genomic stability (Sun et al., 2021). Xue et al. (2020) reported that DNA replication was involved in the genome duplication during the cell cycle. Our findings revealed that GO analysis in KTC-1 was associated with RNA processing, protein binding, cell adhesion, and extracellular exosome. These analyses were performed on the complete set of DEGs, and SLC7A5 was also associated with these biological processes. These findings could provide valuable insights for functional studies (Xue et al., 2020; Shen et al., 2020).

KEGG analysis is used to identify key pathways in thyroid cancer and metabolic diseases. Zhao and Li (2018) performed KEGG analysis and identified the ECM-receptor interaction pathways. These pathways were involved in regulating the cell-matrix adhesion (Zhao and Li, 2018; Xia et al., 2024). Xu H. et al. (2025) reported that PI3K-AKT, MAPK, and Ras signaling pathways were dysregulated in breast, gastric, colorectal, and thyroid cancers (Xu H. et al., 2025; Xia et al., 2024). Our KEGG analysis were enriched with focal adhesion, ECM-receptor interactions, and PI3K-Akt pathways (Nashier et al., 2026). Previous studies reported that dysregulation of these oncogenic pathways could promote the signaling cascades. Therefore, these associated pathways revealed the transcriptional programs in thyroid cancer cells (Derwich et al., 2023; Leandro-García and Landa, 2023).

The protein-protein interaction (PPI) network is widely used to understand protein interactions that regulate biological functions (Khalid et al., 2026). The PPI network was built using the STRING database. Szklarczyk et al. (2025) explored the functional relationships among different proteins through PPI network analysis (Szklarczyk et al., 2025). SLC7A5 was located withthin the network and interacted with TERF1, CARD10, PSAT1, SIRPA, MYC and other different proteins. SPOCK1 showed interaction through mTOR signalling and SLC7A5 also involved in amino acid uptake (Xu et al., 2020; Dunlap et al., 2025). SCARF1 is a glycoprotein involved in disease pathology and SLC7A5 interacts with glycoproteins (Patten, 2018; Scalise et al., 2018). DCDC2 is involved in microtubule-organizing pathways and SL7A5 in tumor microenvironment. Disruption of these processes and pathways causes alterations in protein-protein interactions (Dai et al., 2023; Nachef et al., 2021). These genes interacted with SLC7A5 in the PPI network through regulatory networks. Therefore, these interactions could be helpful in exploring the functional roles of different proteins in biological processes.

This study has some limitations, such as a lack of gene expression validation by RT-qPCR. Patient sample validation and functional validation, such as SLC7A5 knockdown, proliferation assays, amino acid uptake, and mTOR pathway assessment, are further required to establish the biological and clinical significance of SLC7A5.

## 5 Conclusion

In this study, integrated transcriptomics approach revealed the SLC7A5 upregulation in KTC-1. Functional enrichment analyses identified the RNA processing, protein binding, ECM-receptor interactions, and PI3K-Akt. These analyses were performed on the complete set of DEGs, and SLC7A5 was also associated with these biological processes and pathways. Moreover, SLC7A5 showed interaction with different proteins in the PPI network. Future studies are required to explore the functional validation and targeted therapy.

## Data Availability

The original contributions presented in the study are publicly available. This data can be found in the NCBI repository with the BioProject accession number PRJNA1503035.

## References

[B1] Byun H. Lee H. S. Song Y. S. Park Y. J. (2025). Transcriptome of anaplastic thyroid cancer reveals two molecular subtypes with distinct tumor microenvironment and prognosis. Thyroid 35 (4), 367–378. 10.1089/thy.2024.0266 39869083

[B2] Chen X. Wang Z. Zhao X. Zhang L. Zhou L. Li X. (2022). STAT5A modulates CDYL2/SLC7A6 pathway to inhibit the proliferation and invasion of hepatocellular carcinoma by targeting to mTORC1. Oncogene 41 (17), 2492–2504. 10.1038/s41388-022-02273-2 35314791

[B3] Chiba T. (2024). Molecular pathology of thyroid tumors: essential points to comprehend regarding the latest WHO classification. Biomedicines 12 (4), 712. 10.3390/biomedicines12040712 38672067 PMC11048493

[B4] Cleere E. F. Prunty S. O'Neill J. P. (2024). Anaplastic thyroid cancer: improved understanding of what remains a deadly disease. Surg. 22 (1), e48–e53. 10.1016/j.surge.2023.10.002 37866980

[B5] Codrich M. Biasotto A. D’Aurizio F. (2025). Circulating biomarkers of thyroid cancer: an appraisal. J. Clin. Med. 14 (5), 1582. 10.3390/jcm14051582 40095491 PMC11900207

[B6] Patten D. A. (2018). SCARF1: a multifaceted, yet largely understudied, scavenger receptor. Inflamm. Res. 67 (8), 627–632. 10.1007/s00011-018-1154-7 29725698 PMC6028831

[B7] Dai W. Feng J. Hu X. Chen Y. Gu Q. Gong W. (2021). SLC7A7 is a prognostic biomarker correlated with immune infiltrates in non-small cell lung cancer. Cancer Cell Int. 21 (1), 106. 10.1186/s12935-021-01781-7 33632211 PMC7905560

[B8] Dai W. Liu Y. Zhang T. Huang Z. Xu X. Zhao Z. (2023). Spindle function and Wnt pathway inhibition by PBX1 to suppress tumor progression *via* downregulating DCDC2 in colorectal cancer. Oncogenesis 12 (1), 3. 10.1038/s41389-023-00448-4 36739270 PMC9899229

[B9] DunlapK. N. BenderA. BowlesA. BottA. J. TayJ. GrossmannA. H. (2025). SLC7A5 is required for cancer cell growth under arginine-limited conditions. Cell Rep. 44 (1), 115130. 10.1016/j.celrep.2024.115130 39756034

[B10] Fan R. Dong L. Li P. Wang X. Chen X. (2021). Integrated bioinformatics analysis and screening of hub genes in papillary thyroid carcinoma. Plos One 16 (6), e0251962. 10.1371/journal.pone.0251962 34115774 PMC8195368

[B11] Gil-Bernabé S. García-DeLaFuente L. García-Rostán G. (2025). The revolution of targeted therapies in thyroid cancer treatment: present and future promising anti-cancer drugs. Int. J. Mol. Sci. 26 (8), 3663. 10.3390/ijms26083663 40332222 PMC12027515

[B12] Gong W. Chen Y. Zhang Y. (2021). Prognostic and clinical significance of Solute Carrier Family 7 Member 1 in ovarian cancer. Transl. Cancer Res. 10 (2), 602–612. 10.21037/tcr-20-2744 35116394 PMC8797851

[B13] Hosseinzadeh S. Pakizehkar S. Golab-Ghadaksaz H. Hoghooghi Rad L. Hedayati M. (2025). New developments in epigenetic and genetic biomarkers for differentiated thyroid cancer (DTC): moving toward accurate diagnosis and treatment by 2025. Biomed. Adv. 2 (3), 131–136. 10.34172/bma.26

[B14] Jeckelmann J.-M. Zaugg J. Morozova V. Müller J. Kantipudi S. Schroeder M. (2022). Structure, function and pharmacology of SLC7 family members and homologues. Chimia 76 (12), 1011–1018. 10.2533/chimia.2022.1011 38069796

[B15] Karaman E. Yay F. Ayan D. Bayram E. Erturk S. (2025). The clinopathological and prognostic significance of SPOCK1 in gynecological cancers: a bioinformatics based analysis. Biology 14 (2), 209. 10.3390/biology14020209 40001977 PMC11852031

[B16] Khalid Z. Khan K. Nazir F. Yao C. Mehmandousti S. M. M. Shang Y. (2026). Identification and validation of hub genes involved in papillary thyroid carcinoma progression. BMC Cancer 26 (1), 536. 10.1186/s12885-026-15841-6 41851690 PMC13126906

[B17] Leandro-García L. J. Landa I. (2023). Mechanistic insights of thyroid cancer progression. Endocrinology 164 (9), bqad118. 10.1210/endocr/bqad118 37503738 PMC10403681

[B18] Lee P. S. Tseng C. L. Chen J. Y. Chen H. S. Huang C. J. (2026). Clinical characteristics and outcomes of clinically detected versus imaging-detected incidental thyroid cancer. Endocr. Pract. 32 (5), 769–778. 10.1016/j.eprac.2026.02.005 41687737

[B19] Li M. Dal Maso L. Pizzato M. Rumgay H. Vaccarella S. (2026). Thyroid cancer in adolescents and young adults: a population-based study in 185 countries worldwide. Lancet Diabetes & Endocrinol. 14 (2), 112–122. 10.1016/s2213-8587(25)00289-x 41274302

[B20] Menyhart O. Kothalawala W. J. Győrffy B. (2025). A gene set enrichment analysis for cancer hallmarks. J. Pharm. Analysis 15 (5), 101065. 10.1016/j.jpha.2024.101065 PMC12151186 40496069

[B21] Moposita Molina F. D. Agnes G. Zandoná M. R. Izquierdo R. Bilhar F. A. Eidt L. B. (2025). Prevalence and diagnostic reliability of BRAF, RAS mutations, and RET/PTC rearrangements in a Latin American public health service population with thyroid nodular disease. PLoS One 20 (8), e0329407. 10.1371/journal.pone.0329407 40748901 PMC12316217

[B22] Moreno F. Reyes C. Pineda C. A. Castellanos G. Cálix F. Calderón J. (2022). Anaplastic thyroid carcinoma with unusual long-term survival: a case report. J. Med. Case Rep. 16 (1), 39. 10.1186/s13256-021-03249-8 35101107 PMC8805419

[B23] Nachef M. Ali A. K. Almutairi S. M. Lee S. H. (2021). Targeting SLC1A5 and SLC3A2/SLC7A5 as a potential strategy to strengthen anti-tumor immunity in the tumor microenvironment. Front. Immunology 12, 624324. 10.3389/fimmu.2021.624324 33953707 PMC8089370

[B24] Nashier D. Manjunath G. K. Parveen A. Malik S. Mitra T. Goswami C. (2026). Decoding the mutational hierarchy of thyroid cancer and associated signaling pathways. Archives Med. Res. 57 (4), 103366. 10.1016/j.arcmed.2025.103366 41494277

[B25] Pan L. Zhang L. Fu J. Shen K. Zhang G. (2024). Integrated transcriptome sequencing and weighted gene co-expression network analysis reveals key genes of papillary thyroid carcinomas. Heliyon 10 (7), e27928. 10.1016/j.heliyon.2024.e27928 38560266 PMC10981042

[B26] Qiu J. Zhang W. Xia Q. Liu F. Li L. Zhao S. (2016). RNA sequencing identifies crucial genes in papillary thyroid carcinoma (PTC) progression. Exp. Molecular Pathology 100 (1), 151–159. 10.1016/j.yexmp.2015.12.011 26708423

[B27] Sarin K. Bhagat R. Punia R. P. S. Das A. Handa U. (2022). Diagnostic role of Galectin-3 immunohistochemistry in thyroid lesions. J. Head & Neck Physicians Surg. 10 (2), 152–156. 10.4103/jhnps.jhnps_37_22

[B28] Scalise M. Galluccio M. Console L. Pochini L. Indiveri C. (2018). The human SLC7A5 (LAT1): the intriguing histidine/large neutral amino acid transporter and its relevance to human health. Front. Chemistry 6, 371668. 10.3389/fchem.2018.00243 29988369 PMC6023973

[B29] Derwich A. Sykutera M. Bromińska B. Andrusiewicz M. Ruchała M. Sawicka-Gutaj N. (2023). Clinical implications of mTOR expression in papillary thyroid cancer—A systematic review. Cancers 15 (6), 1665. 10.3390/cancers15061665 36980552 PMC10046096

[B30] Shen Y. Dong S. Liu J. Zhang L. Zhang J. Zhou H. (2020). Identification of potential biomarkers for thyroid cancer using bioinformatics strategy: a study based on GEO datasets. BioMed Research International 2020 (1), 9710421. 10.1155/2020/9710421 32337286 PMC7152968

[B31] Sokolov A. M. Holmberg J. C. Feliciano D. M. (2020). The amino acid transporter Slc7a5 regulates the mTOR pathway and is required for granule cell development. Hum. Molecular Genetics 29 (18), 3003–3013. 10.1093/hmg/ddaa186 32821949 PMC7645712

[B32] Sun T. Guan Q. Wang Y. Qian K. Sun W. Ji Q. (2021). Identification of differentially expressed genes and signaling pathways in papillary thyroid cancer: a study based on integrated microarray and bioinformatics analysis. Gland. Surg. 10 (2), 629–644. 10.21037/gs-20-673 33708546 PMC7944059

[B33] Szklarczyk D. Nastou K. Koutrouli M. Kirsch R. Mehryary F. Hachilif R. (2025). The STRING database in 2025: protein networks with directionality of regulation. Nucleic Acids Research 53 (D1), D730–D737. 10.1093/nar/gkae1113 39558183 PMC11701646

[B34] Vaio F. Moliterni C. Mardente S. Misasi R. Mari E. (2026). State of the art on thyroid cancer biology and oncology. Biomedicines 14 (1), 168. 10.3390/biomedicines14010168 41595702 PMC12838939

[B35] Wang L. Zhang Y. Yang J. Liu L. Yao B. Tian Z. (2021). The knockdown of ETV4 inhibits the papillary thyroid cancer development by promoting ferroptosis upon SLC7A11 downregulation. Dna Cell Biology 40 (9), 1211–1221. 10.1089/dna.2021.0216 34283663

[B36] Wang F. Zhang S. Chen Z. Gu X. Zhang G. Zhang H. (2024). N7-methyladenosine-induced SLC7A7 serves as a prognostic biomarker in pan-cancer and promotes CRC progression in colorectal cancer. Sci. Rep. 14 (1), 30755. 10.1038/s41598-024-80885-2 39730571 PMC11680768

[B37] Xia J. Shi Y. Chen X. (2024). New insights into the mechanisms of the extracellular matrix and its therapeutic potential in anaplastic thyroid carcinoma. Sci. Rep. 14 (1), 20977. 10.1038/s41598-024-72020-y 39251678 PMC11384763

[B38] Xu M. Zhang X. Zhang S. Piao J. Yang Y. Wang X. (2020). SPOCK1/SIX1axis promotes breast cancer progression by activating AKT/mTOR signaling. Aging (Albany NY) 13 (1), 1032. 10.18632/aging.202231 33293473 PMC7835061

[B39] Xu T. Zhang X. Chen Q. Yang C. Deng B. Armstrong D. G. (2025a). SLC7 transporters at the crossroads of amino acid metabolism and diabetes pathophysiology: insights and therapeutic perspectives. Front. Nutr. 12, 1467057. 10.3389/fnut.2025.1467057 40469683 PMC12133496

[B40] Xu H. Ren S. M. Wang Y. Zhang T. T. Lu J. (2025b). Abnormal activation of the Ras/MAPK signaling pathway in oncogenesis and progression. Cancer Adv. 8, e25002. 10.53388/2025825002

[B41] Xue G. Lin X. Wu J. F. Pei D. Wang D. M. Zhang J. (2020). Identification of key genes of papillary thyroid carcinoma by integrated bioinformatics analysis. Biosci. Reports 40 (8), BSR20201555. 10.1042/BSR20201555 32766727 PMC7433002

[B42] Zhao H. Li H. (2018). Network-based meta-analysis in the identification of biomarkers for papillary thyroid cancer. Gene 661, 160–168. 10.1016/j.gene.2018.03.096 29625265

